# An H_2_S Sensor Based on Electrochemistry for Chicken Coops

**DOI:** 10.3390/s16091398

**Published:** 2016-08-31

**Authors:** Lihua Zeng, Mei He, Huihui Yu, Daoliang Li

**Affiliations:** 1 Beijing Engineering and Technology Research Center for Internet of Things in Agriculture, China Agricultural University, Beijing 100083, China; zlh52103@gmail.com (L.Z.); lll333@163.com (M.H.); yhh1990@126.com (H.Y.); 2 College of Mechanical and Electrical Engineering, Agricultural University of Hebei, Baoding 071001, China; 3 Key Laboratory of Agricultural Information Acquisition Technology, Ministry of Agriculture, China Agricultural University, Beijing 100083, China

**Keywords:** coop, H_2_S gas, sensor

## Abstract

The recent modernization of the livestock industry lags behind the scale of the livestock industry, particularly in indoor environmental monitoring. In particular, the H_2_S gas concentration in chicken coops affects the growth and reproductive capacity of the chickens and threatens their health. Therefore, the research and development of a low-cost, environmentally friendly sensor that can achieve on-line monitoring of H_2_S gas has a notably important practical significance. This paper reports the design of an H_2_S gas sensor, with selection of an electrochemical probe with high accuracy and wide measurement range using the relatively mature technology of electrochemical sensors. Although the probe of the sensor is the main factor that affects the sensor accuracy, the probe must be combined with a specifically designed signal condition circuit that can overcome the lack of an electrode to satisfy the requirements for the interconnection and matching between the output signal and the test instrument. Because the output current of the electrochemical electrode is small and likely to be disturbed by noise, we designed signal-conditioning modules. Through the signal-conditioning circuit, the output signal of the current electrode can be converted into a voltage and amplified. In addition, we designed a power control module because a bias voltage is necessary for the electrode. Finally, after the calibration experiment, the accurate concentration of H_2_S gas can be measured. Based on the experimental analysis, the sensor shows good linearity and selectivity, comparatively high sensitivity, perfect stability and an extremely long operating life of up to two years.

## 1. Introduction

In recent years, livestock production has become an industrial-scale process, where several thousand cattle or pigs and 100,000 or more chickens are fed grains and produced in a single facility [1]. Because the scale of production and intensification of the livestock industry continues to increase, environmental control is always a concern issue for livestock producers. In fact, various toxic gases are produced in the coops because of increased breeding density and the closed environment. In addition, the monitoring and control for H_2_S can easily be neglected because of the generally low H_2_S gas concentration in chicken coops. Long-term living in the environment with malodorous gases causes livestock to grow slowly, affects livestock breeding and increases the susceptibility of air-spread disease. The health of humans and livestock in an environment with a long-term low H_2_S gas concentration can be seriously affected [2]. Therefore, effective monitoring of the H_2_S gas concentration in animal housing is important to reduce agricultural pollution and improve the safety of livestock production [3]. Thus, the research and development of an H_2_S gas sensor that is low cost, environmentally friendly and able to achieve on-line monitoring of H_2_S gas is highly significant in practice [4].

There have been a growing number of studies on the monitoring of H_2_S gas, and researchers have provided many detection methods [5]. Reported methods for H_2_S gas detection mainly include semiconductor sensors, electrochemical sensors and optical sensors [6]. Various solid-state hydrogen sulfide sensors based on semiconductor metal oxides such as SnO_2_, ZrO_2_, CeO_2_, and WO_3_ have been reported in the literature [7]. Recently, many studies focused on nanocrystalline copper [8,9,10] and CuO modified layers [11,12,13,14]. With the CuO modified SnO_2_, the selectively and sensitivity were promoted [15,16]. Semiconductor sensors are simple, have good stability and have been widely applied in industrial, military, agricultural and other fields with rapid development. The gases under test usually must be heated to a certain temperature to promote the interaction with the semiconductor materials. The optical gas sensor is also generally used to detect H_2_S gas. It has the highest accuracy, which can detect an H_2_S gas concentration below 1 ppm, and a short reaction time [17,18]. However, its principle is complex, and the optical system requires a complete system [19]. The expensive price and large volume of optical gas sensors make the application of optical gas sensors in portable systems difficult.

After Carl Wagner’s interpretation of zirconia, many advances in chemical sensor applications have appeared. In general, the electrochemical techniques for chemical measurements have several major advantages compared to other methods [20]. Compared with semiconductor gas sensors and optical gas sensors, electrochemical gas sensors have an inexpensive price and high gas selectivity, which simplifies the H_2_S gas detection. Electrochemical gas sensors produce electrical signals by the chemical oxidation or reduction reaction of certain gases in the air according to the electrolytic cell principle, and the gas concentration is obtained by detecting the magnitude of the current response [21]. Electrochemical gas sensors are frequently used to detect malodorous gases in animal housing. Electrochemical sensors have good gas selectivity by the catalysts and a selective permeability membrane, which can detect the concentration of a specific gas in a gas mixture. Electrochemical sensors are usually used to detect H_2_S, NH_3_ and other gases emitted from animal housing. Because of the small size and fast response time, electrochemical gas sensors are often used in real-time monitoring of malodorous gases in animal housing [22]. To avoid problems because of the electrolyte solution, studies have examined solid electrolytes. At present, organic gel electrolyte gas sensors, solid polymer electrolyte gas sensors and other products are available. Researchers at Wuhan University developed all-solid controlled-potential electrolysis oxygen sensors using the Nafion membrane [23]. However, the conductivity of the Nafion membrane is susceptible to water, so the sensor is restricted by the environmental humidity in the field. Some researchers add a water tank that can adjust the humidity of the environment in the sensors to solve the dependence of the Nafion membrane conductivity on water, which is obviously not conducive to sensor miniaturization. The technology of solid electrolytes is a promising distributed technology, but it has not been widely used due to its immaturity and faultiness.

This paper designs an H_2_S gas sensor based on electrochemical electrodes that can be used in chicken coops. Although there are commercially available electrochemical sensors, which have high precision, high sensitivity and wide linear range, the specific H_2_S sensors for chicken coops are not available. Because of the characteristics of electrochemical electrodes, which include low power and vulnerability to interference, and the three-electrode system, we designed a constant-potential control circuit, amplifying circuit and zero-adjustment circuit. After a calibration experiment, H_2_S gas can be accurately measured. Based on the experimental analysis, the sensors show good linearity and selectivity, comparatively high sensitivity, perfect stability and an extremely long operating life of up to two years.

## 2. Materials and Methods

### 2.1. Electrochemistry

Electrochemical gas sensors produce electrical signals by the chemical oxidation or reduction reaction of certain gases in the air according to the electrolytic cell principle. The gas concentration is obtained by detecting the magnitude of the current response. Constant-potential electrolysis cell gas sensors consist of three electrodes and an electrolyte.

The oxidation reaction of hydrogen sulfide on the working electrode is as follows:

H_2_S + 4H_2_O → SO_4_^2−^+ 10H^+^ + 8e^−^ (1)

The reduction reaction of hydrogen sulfide oxidation on the counter electrode is as follows:

O_2_ + 2H^+^ + 2e^−^ → H_2_O (2)

The overall reaction of hydrogen sulfide oxidation is as follows:

H_2_S + 2O_2_ → H_2_SO_4_ (3)

With these reactions, the redox reactions produce positrons and electrons on the counter electrode and working electrode, and the electrons form a current under the effect of an electric field. The current value between the counter electrode and the working electrode is defined by the diffusion kinetic equation

(4) $$I=\frac{nFADC}{\delta }$$

where *n* is the number of electrons in the process, *F* is Faraday constant, *A* is the active surface of the sensitive electrode, *D* is the gas diffusion coefficient, *δ* is the thickness of the gas diffusion membrane, and *C* is the gas concentration. These parameters are constant, whereas the electrode is fixed. Current *I* is proportional to H_2_S gas concentration *C* as long as the size of the measured current *I* can launch the H_2_S gas concentration *C*.

### 2.2. Materials and Experiments

A three-electrode system was introduced to test the performance. Pt was used as the counter electrode, working electrode, calomel electrode and reference electrode; a concentrated sulfuric acid solution with additives was used as the liquid electrolyte [24]. The liquid electrolyte included sulfuric acid (10%–28%), NaCl (0.5%–1.5%), Glycerin (1%–3%) and Hexamethylenetetramine (0.1%–0.5%).

Some experiments were performed in a laboratory to evaluate the characteristics of the sensor before use in the field, such as the linearity, accuracy, repeatability, stability, and response time-to-recovery. The desired concentrations were diluted with a standard hydrogen sulfide gas (1% H_2_S nitrogen fill). A gas mixer (GSM-3 Gas Mixer, CWE Inc., Ardmore, PA, USA) was used to mix the hydrogen sulfide and N_2_.

The field experiment was performed in a caged-hen layer house, which was 28 m long, 8 m wide, and 3 m high with a three-layer cascade cage and four inner cages in two columns [25]. The typical range of temperature was 15–32 °C and varying humidity, relative humidity (RH) was 25%–65%. The concentration of H_2_S was not more than 8 mg/L, and always in the range of 1–5 mg/L. To monitor the concentration, the designed H_2_S sensors were placed in each layer of the chicken cage.

### 2.3. Sensor Set-up

**Figure 1:**
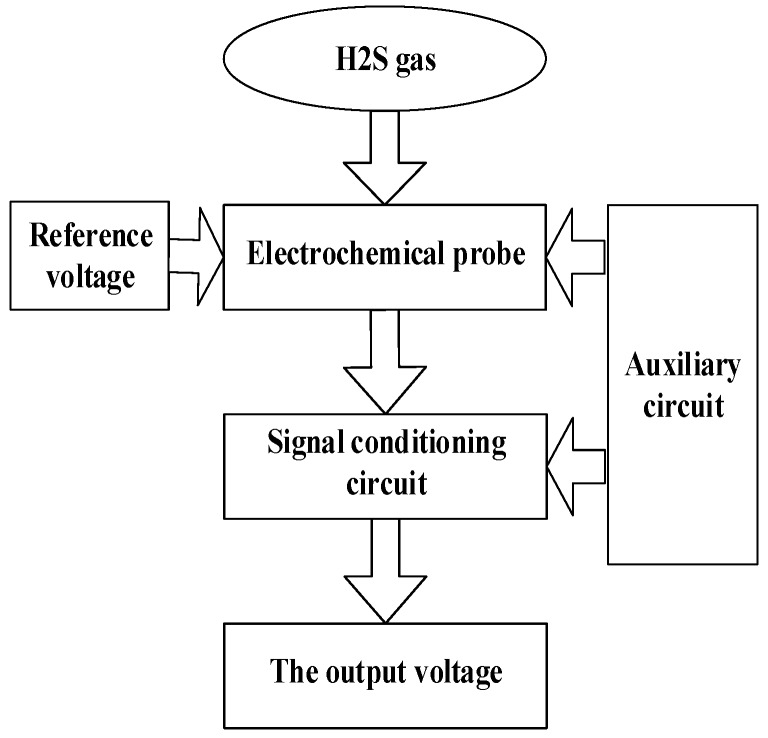
Measurement Circuit Design Diagram of the Sensor.

Through the analysis of the working principle of electrochemical gas sensors, a design diagram of the measurement circuit that detects the H_2_S gas concentration is presented. The measurement circuit design diagram of the sensor is shown in Figure 1.

The sensor circuit is mainly composed of four parts:

(1) Three-electrode sensor: It can directly contact hydrogen sulfide gas and convert the hydrogen sulfide gas concentration to a proportional current.
(2) Bias circuit: It can provide a stable voltage for the sensors to control the electrochemical reaction.
(3) Signal-conditioning circuit: It can convert the electrical signal, which is proportional to the gas concentration, into a voltage signal and amplify the voltage signal, which is easily measured by the microcontroller.
(4) Auxiliary circuit: It includes the power supply circuits and peripheral circuits.

#### 2.3.1. Signal-Conditioning Modules

(1) Current-voltage conversion circuit

Because the output of the electrode is a current signal, which is proportional to the gas concentration, the current-voltage conversion circuit can convert the output current signal back into a voltage signal, which is easily connected with the measuring instruments.

In any case, we must ensure that the *R*13 voltage is less than 10 mV; otherwise, the performance of the sensor is adversely affected. Low-resistance sensors can obtain faster responses; even in this circuit, the resistance can be 0. However, a smaller value can achieve a better balance between the response time and the noise, which can also reduce the humidity and instant effect. Thus, we selected a 0.1% precision resistor for *R*13.

This circuit includes an inversely proportional arithmetic circuit with a low input resistance and low demand for the common mode rejection ratio for the current-voltage conversion. This circuit enables the selection of gain within certain limits by adjusting a single resistor *R*6. The transfer function of this amplifier is

*V*_out_1 = *R*6 × *I*_in_ (5)

(2) Output voltage amplifier

In the detection process of a weak signal, to reduce the interference of the integrated operational amplifier, we should select the ideal op-amp IC for the circuit. The main parameters of the chip must be: a small input bias current, a small input offset voltage, a small drift, a large common-mode rejection ratio and a large input resistance. Currently, many integrated operational amplifiers are suitable for these conditions, such as: AD8571, LMC6482, LF351 and OPA2703 (Texas Instruments, Dallas, TX, USA), and this design used the dual operational amplifier LMC6482.

Assuming that this operational amplifier is an ideal op-amp, the two-input voltage difference of the operational amplifier is almost zero because of its infinite open-loop gain. When the matching and op amp are perfect, the transfer function for this amplifier is

*V*_out_ = ((*R*9/*R*21) + 1) × *V*_out_1 (7)

Resistor *R*9 is used to feedback the output to the inverting input of the op amp. The circuit used the RC lag compensation to connect the feedback resistor and capacitors in parallel, which offsets the effects of the input capacitance and breaks the phase balance of self-oscillation to eliminate self-oscillation in the amplifier. A resistor that connects the output terminal of the Op-amp is used to ensure the output short-circuit.

#### 2.3.2. Constant Voltage Control Module for the Reference Electrode

**Figure 2:**
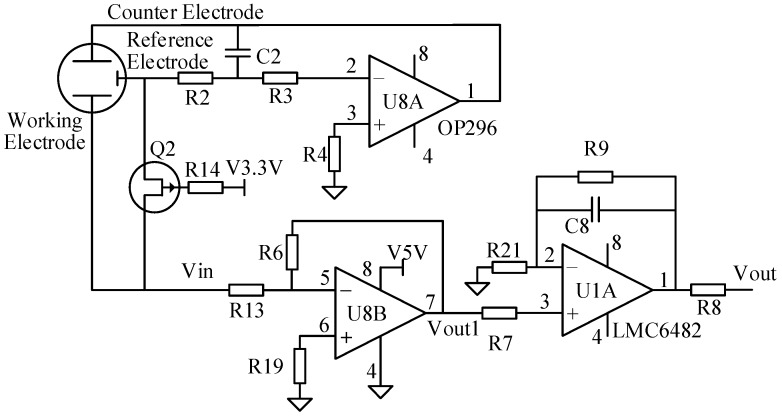
Measurement Circuit and Signal Conditioning Circuits of the Sensor.

Because the selection bias of the electrochemical gas sensor determines the type of measured gases and the bias stability has a direct effect on sensitivity and noise, the design of the constant-voltage control circuit makes the sensor reduce noise and improves the ability to select the measured gas while removing other gases from the measurement. As shown in Figure 2, the main objective of the circuit is to maintain a stable voltage between the reference electrode and the working electrode to control the electrochemical reaction and provide a proportional current to the output of the sensor signal.

When the switch is open, U8-A can only have a small bias voltage (<100 mV). Otherwise, the amplifier will make the sensor have a large bias, which makes the sensor require a long period of stability after a short time.

The extremely low power requirements and guaranteed operation from 3 V to 12 V of the OP296 chip make these amplifiers perfectly suited to monitor the battery usage and control battery charging.

To extend the life of the probe, when there is no power on the reference electrodes, the sensors must be shorted. The working electrode and a reference electrode are connected with a Field Effect Transistor (FET), which is disconnected when the probe is powered.

## 3. Results

A specific dynamic test system for the gas sensors was used in conjunction with the standard gas device to produce a volume fraction of standard H_2_S gas. The detection of the final H_2_S concentrations by the gas detector pump was observed.

### 3.1. Calibration of H_2_S Gas Sensor

We use standard gas bottles for the hydrogen sulfide gas based on the controlled release of the cylinder valve of hydrogen sulfide gas and purge time. We opened the valve 8 times; the gas concentrations increased, and we used the sensors to measure 8 times to obtain 8 different concentrations in the measured data.

To reduce the measurement error of gas diffusion of the sensor, the gas concentration was simultaneously measured by the designed sensor and handheld sensors. From these measurements, we obtained the data shown in Table 1.

**Figure 3:**
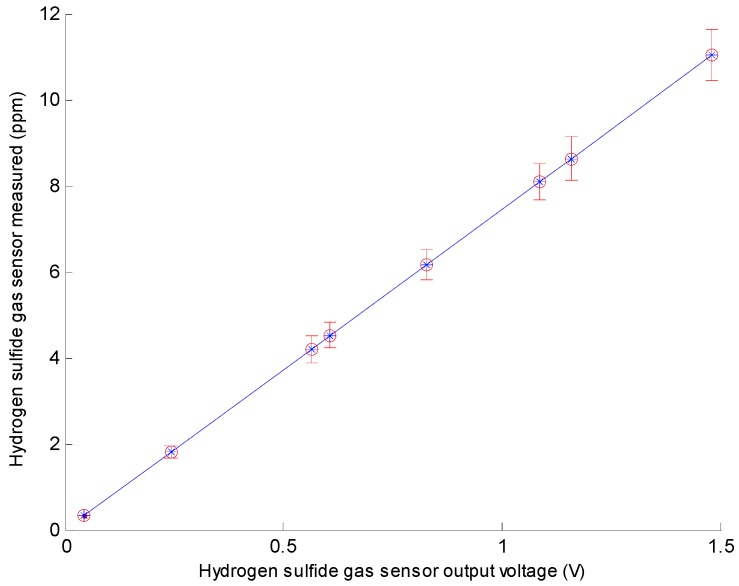
Hydrogen Sulfide Gas Sensor Calibration Curve.

Using the data shown in Table 1, we obtained a calibration curve of the gas sensor. Figure 3 shows the corresponding H_2_S gas outputs of the sensors for a 3.3 V operating voltage, 25 ± 1 °C environmental temperature, and 30% ± 3% RH. From the characteristic curve, we obtained the calibration curve *y* = 7.4411*x* + 0.0005, as shown in Figure 3, based on these data. *R*2 of the curve is 1; hence, the linearity of the pH sensor is very good. Because the output is a linear graph of *y* = A*x* + B, we could identify A and B after the calibration.

### 3.2. Analysis of the Sensor Performance

#### 3.2.1. Accuracy Analysis

We prepared 3 gas samples by controlling the standard gas bottle valve pressure and breathing time at room temperature. The concentration of the gas sample was set to 3 ppm, 5 ppm and 7 ppm. The sensor was placed in a sealed box, and each sample was measured 8 times. The measurement results are shown in Table 2. The data analysis indicates that the accuracy of the sensor is very good because all relative errors of the sample gas were within ±10%. From the data in Table 1, we find that this sensor measured a smaller gas concentration than the standard concentration of a handheld meter. The errors can be attributed to the suction pump of the handheld meter, which can gather H_2_S gas, whereas the sensor cannot.

#### 3.2.2. Repeatability

**Figure 4:**
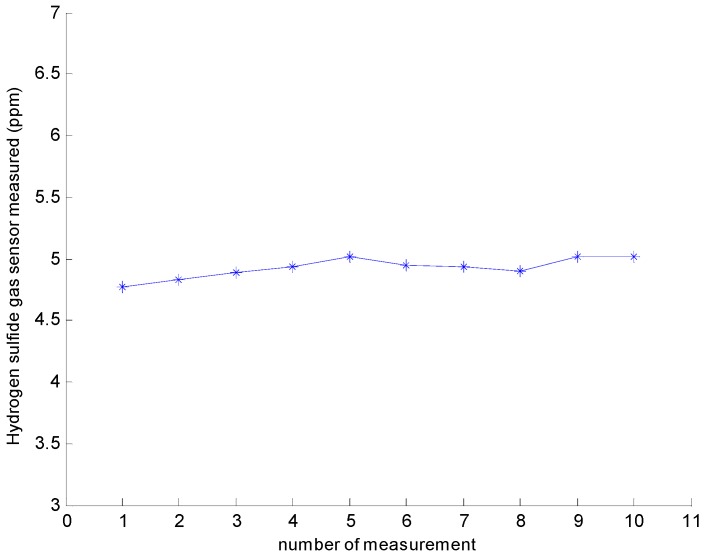
Repeatability of the Measurements.

The gas sensor repeatability test was performed 10 times with an H_2_S gas concentration of 5 ppm. The results are shown in Figure 4. From the test results, we observe that the sensor output fluctuated within 5 ppm. The relative standard deviation (RSD) was 1.67%. The smaller RSD implies that the determination results were more concentrated, and the precision was better. The results show that the sensor has high repeatability.

#### 3.2.3. Stability

We used 5.00 and 6.00 ppm of hydrogen sulfide gas. We put the sensor into the sample gas to measure the stability of the H_2_S gas sensor. The data were recorded every hour and continuously measured ten times. The results are shown in Table 3. The data analysis result indicates that the stability of the sensor is good, and the measured values are in the range of error.

#### 3.2.4. Response Time-to-Recovery

**Figure 5:**
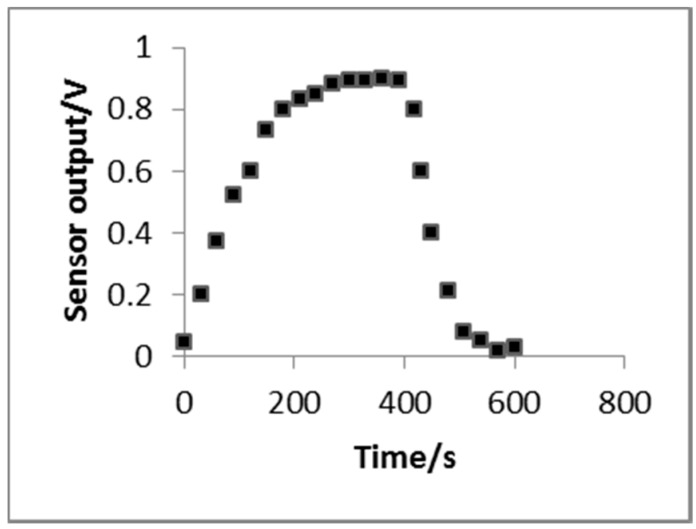
Sensor response curve vs. recovery time.

The response time-to-recovery test of the H_2_S gas sensor used a dedicated dynamic test system. The sensor output accessed the data capture card of the PC, and the gas density using a 5-ppm volume fraction. The results are shown in Figure 5. The test results show that when the H_2_S gas sensor experiences a 0.9 V output, the recovery time is approximately 1 min. The response time and recovery time were longer than those of commercial H_2_S sensors. However, the H_2_S concentration in chicken coops always slowly changes, and the response time-to-recovery can satisfy the monitoring requirement.

### 3.3. Discussion

As mentioned in the introduction, semiconductor sensors [26] and optical sensors are research hotspots for H_2_S detection. Similar to the results of this paper, some studies by Tiexiang Fu developed a semiconductor sensor that could independently detect H_2_S only at concentrations greater than 370 ppm, which showed a good repeatability of below 4.4% (in terms of relative standard deviation (RSD)) with a notably fast response of 8 s in the concentration range of 100–540 ppm. In another study, G.H. Jain designed a semiconduction sensor that could measure H_2_S gas in the 1–100 ppm range (in the presence of air) with an estimated LOD of 4–10 ppb [27]. However, it only recovered after heating to 200 °C. Compared with the results of other authors, the accuracy of the reported sensor in this paper is consistent with other semiconductor sensors. In another similar study, Donglin Tang designed an optic sensor that could achieve a detection limit of approximately 10 ppm [28], but the structure of this sensor was too complex to produce at low cost. In addition, an electrochemical sensor was developed by Hossein Dehnavi [29], but this sensor could not determine the present concentration of the gas. Thus, the design of an H_2_S gas sensor based on electrochemistry is the most appropriate for the detection of H_2_S gas in chicken coops.

In addition, we performed some experiments and analyzed the important parameters while focusing on the problems that might arise. We found that the gas concentration measured by this sensor was less than the standard concentration when a handheld meter was used. The errors can be attributed to the suction pump of the handheld meter, which can gather the H_2_S gas, whereas the sensor cannot. However, the current accuracy of the sensor satisfies the required accuracy of sensors in animal housing. Because of the principle of electrochemistry, the response time of the sensor is longer than other sensors such as the optic sensor. However, changing the diffusion barrier enables the sensor manufacturer to tailor the sensor to a particular target gas concentration range. In addition, because the diffusion barrier is primarily mechanical, the calibration of electrochemical sensors tends to be more stable over time, and thus electrochemical-sensor-based instruments require much less maintenance than other detection technologies. Furthermore, the device is much smaller than other sensors, and the cost of the electrochemical sensor is acceptable compared with the optic sensor.

## 4. Conclusions

In this paper, a H_2_S gas sensor based on electrochemistry for chicken coops, which is a low-cost, low-power option that is able to monitor the concentration of H_2_S gas in the field, is designed considering the existing problems in the H_2_S gas measurement. According to the characteristics of the hydrogen sulfide gas concentration in chicken coops, an electrochemical hydrogen sulfide electrode was produced, and we designed a power management module based on the required work for the offset voltage that the electrode needed. Concurrently, this paper designs the output signal-conditioning module with a simple and stable circuit to transform the output current signal from the electrode into a voltage and amplify the signal. After the calibration of the H_2_S gas sensor, the experimental data show that the H_2_S gas sensor has good linearity, accuracy, repeatability and stability. The H_2_S gas sensor shows great potential in gas monitoring in chicken coops and similar applications.

## Figures and Tables

**Table 1:** Experimental Data.

Hydrogen Sulfide Gas Samples	Setting Concentration (ppm)	Hydrogen Sulfide Gas Sensor Output Voltage (V)	Hydrogen Sulfide Gas Sensor Measured (ppm)
1	0	0.044	0.327
2	2	0.242	1.800
3	4	0.565	4.204
4	5	0.607	4.516
5	6	0.827	6.153
6	8	1.087	8.088
7	9	1.159	8.624
8	11	1.482	11.027

**Table 2:** Accuracy of the Measurements.

Sample	Result (ppm)								The Average (ppm)	Absolute Error (ppm)	Relative Tolerance
	1	2	3	4	5	6	7	8			
3	2.669	2.721	2.863	2.932	2.993	2.921	2.901	2.899	2.862	0.078	2.72%
5	4.771	4.825	4.892	4.931	5.012	4.952	4.938	4.901	4.903	0.054	1.10%
7	6.665	6.891	6.937	6.991	7.002	7.159	7.013	7.059	6.965	0.083	1.19%

**Table 3:** Stability of the Measurements.

Sample	Measurement Result (ppm)										Average Value	Fractional Error
	1	2	3	4	5	6	7	8	9	10		
5.00	4.86	4.79	4.89	4.93	4.89	4.91	5.09	4.98	5.12	5.10	4.96	2.68%
6.00	6.12	6.09	6.16	6.11	6.13	6.01	5.90	5.92	6.06	6.10	6.06	1.17%
